# The plastome and phylogenetic status of *Cotoneaster rosiflorus* (Rosaceae)

**DOI:** 10.1080/23802359.2024.2385616

**Published:** 2024-08-02

**Authors:** Kaikai Meng, Qiang Fan, Min Lin, Shouhui Huang

**Affiliations:** a Guangxi Key Laboratory of Quality and Safety Control for Subtropical Fruits, Guangxi Subtropical Crops Research Institute, Nanning, China; b Key Laboratory of Quality and Safety Control for Subtropical Fruit and Vegetable, Ministry of Agriculture and Rural Affairs, Guangxi Subtropical Crops Research Institute, Nanning, China; c State Key Laboratory of Biocontrol and Guangdong Provincial Key Laboratory of Plant Resources, School of Life Sciences, Sun Yat-sen University, Guangzhou, China

**Keywords:** *Cotoneaster*, chloroplast genome, phylogenomics, chloroplast capture

## Abstract

Endemic to Taiwan Province, China, *Cotoneaster rosiflorus* Kun-Cheng Chang & Fu-Yuan Lu 2011 (Rosaceae) holds significant ecological and ornamental importance. Despite its value, research on its molecular data and phylogenetic position has remained limited. In this study, we addressed this gap by sequencing the genome-skimming data, assembling its plastome, and investigating its phylogenetic position. The plastome, spanning 159,449 bp in length, consisted of a large single-copy (87,433 bp), a small single-copy (19,262 bp), and two inverted repeat regions (26,377 bp). We annotated a total of 128 functional genes, including 84 protein-coding genes, 36 transfer genes, and eight ribosomal RNA genes. The phylogenetic results indicated that *C. rosiflorus* is closely related to *C. dammerii*, suggesting that *C. rosiflorus* might have captured its chloroplast from *C. dammerii* through hybridization and introgression events. This study offered valuable insights for forthcoming phylogenetic and population genetic investigations of *Cotoneaster*.

## Introduction

According to the monograph of *Cotoneaster*, the genus of *Cotoneaster* comprises up to 370 species, with ubiquitous hybridization, polyploidization, and apomixis, known for its taxonomic complexity (Fryer and Hylmö 2009). According to morphological characters, this genus can be divided into two subgenera, *Chaenopetalum* (mainly with white flowers and spreading petals) and *Orthopetalum* (mostly red or pink flowers and erect petals) (Fryer and Hylmö 2009). The investigation into phylogenetic relationships within this genus relied on a restricted set of gene loci, resulting in low support values and resolution (Li et al. 2014) Recently, the reconstruction of phylogenetic trees based on genome-skimming data has significantly bolstered our understanding, offering a robust framework that clarifies the relationships among these species (Meng et al. 2021).

*Cotoneaster rosiflorus* Kun-Cheng Chang & Fu-Yuan Lu 2011 is endemic to Taiwan Province, China (Chang et al. 2011). As a semi-evergreen creeping shrub, it holds significant ecological and ornamental importance. Notably, it features erect or slightly spreading pink petals, setting it apart from the sympatric species *C. dammerii*, which has spreading white petals. However, previous studies have overlooked molecular data for *C. rosiflorus*. Consequently, we aim to characterize its plastome and ascertain its phylogenetic position.

## Materials and methods

**Figure 1.**
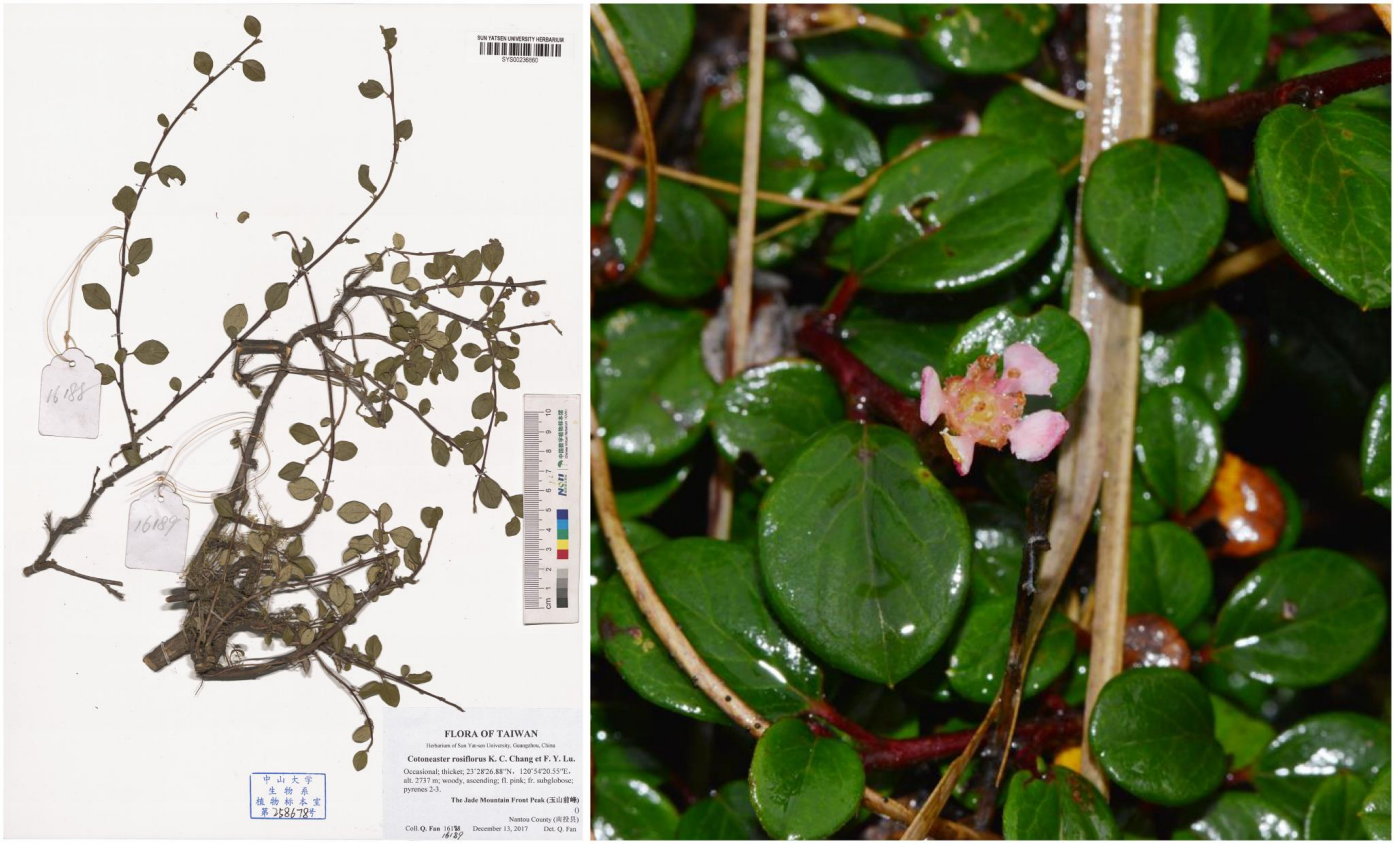
Herbarium specimen and photograph of *Cotoneaster rosiflorus*. Photos were taken by Qiang Fan.

Fresh leaves of one individual were collected from Nantou county, Taiwan province, China (N23°28′27″, E120°54′21″; Altitude: 2737 m) and preserved in desiccant. The voucher specimen was deposited in the herbarium at Sun Yat-sen University (http://lifesciences.sysu.edu.cn/, Wenbo Liao, lsslwb@mail.sysu.edu.cn) under the voucher number *Q. Fan 16189* (Figure 1). The qualified genomic DNA was obtained according to the methods of Meng et al. (2021). A DNA library was constructed and paired-end (2 × 150 bp) high-throughput sequencing was conducted using the HiSeq X Ten system.

Raw reads were processed using fastp v.0.2 (Chen et al. 2018). The plastome was assembled using NOVOPlasty2.6.3 (Dierckxsens et al. 2017) with the plastome (MK614799) and *rbcL* gene of *C. integerrimus*. Annotations were completed using a combination of GeSeq (Tillich et al. 2017) and CPGAVAS2 (Shi et al. 2019).

Totally, 73 plastomes were retrieved from NCBI. These plastomes were aligned and trimmed using MAFFT v7 (Rozewicki et al. 2019) and TrimAl v1.4 (Capella-Gutiérrez et al. 2009). Alignment direction was meticulously reviewed individually, guided by the LAST plot file generated by MAFFT v7 (Rozewicki et al. 2019). The maximum-likelihood phylogeny was reconstructed in IQ-TREE v2.1 (Minh et al. 2020) by running 2,000 replicates of SH-aLRT (SH approximate likelihood ratio test) and UFBS (UltraFast BootStraps) (Hoang et al. 2018) using GTR + F + I as the optimum model.

## Results

**Figure 2.**
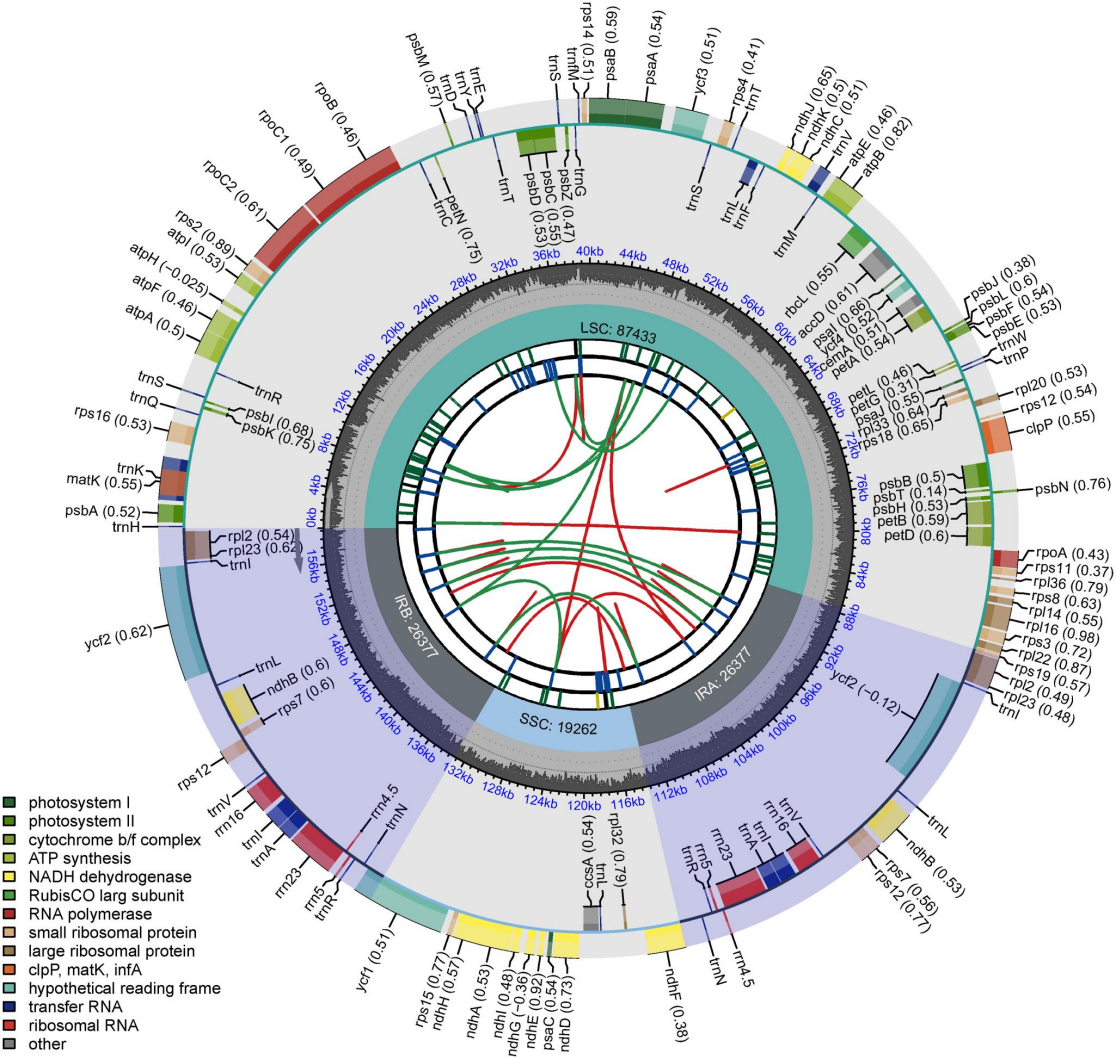
The overall features of plastome of *Cotoneaster rosiflorus*. Tracks from the innermost to outermost indicate the forward (red) and reverse repeats (green), the tandem repeats, the microsatellite sequences, the size of the LSC, SSC and IRs, the GC contents, and the genes. The transcription directions for the inner and outer genes are clockwise and anticlockwise, respectively.

**Figure 3.**
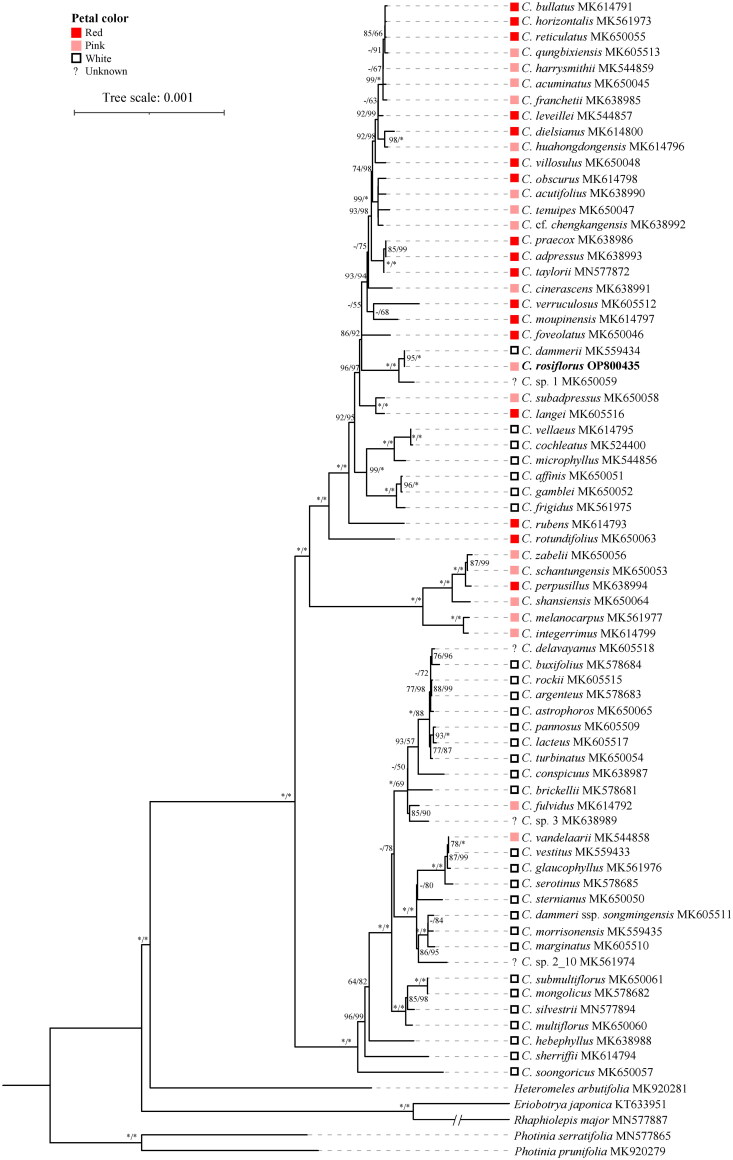
Maximum-likelihood phylogeny of *Cotoneaster*. Numbers indicate SH-aLRT/UFBS and values below 50 are indicated by ‘-’. Specifically, two sequences (MK920279 and MK920281) were downloaded from publicly available database based on Liu et al. (2019), five sequences (MN577865, MN577872, MN577887, MN577894, and KT633951) were obtained from Liu et al. (2020), and the remaining 66 sequences were sourced from Meng et al. (2021).

Approximately 8 Gb of raw data was obtained. The plastome exhibited a length of 159,449 bp. The coverage depth ranged from a minimum of 1,238 to a maximum of 6,934 (Figure S1). Using CPGView (Liu et al. 2023), the circular map, trans-splicing and cis-splicing genes were visualized (Figure 2 & Figure S2). The plastome had a typical quadripartite structure, comprising a large single-copy (87,433 bp), a small single-copy (19,262 bp), and two inverted repeat regions (26,377 bp). The average Guanine-Cytosine content was 63.4%. A total of 128 functional genes were annotated, among which 84 were protein-coding genes, 36 were transfer genes, and eight were ribosomal RNA genes. Seventeen genes were duplicated and located in the IR regions, comprising four rRNAs (*rrn4.5*, *rrn5*, *rrn16*, and *rrn23*), eight tRNAs (*trnA-UGC*, *trnI-CAU*, *trnI-GAU*, *trnL-CAA*, *trnN-GUU*, *trnR-ACG*, *trnS-GCU*, and *trnV-GAC*), and five protein-coding genes (*rpl2*, *rpl23*, *rps7*, *ndhB*, and *ycf2*). Among the protein-coding genes, nine (*atpF*, *rpl2*, *rpl16*, *ndhA*, *ndhB*, *petB*, *petD*, *rpoC1*, and *rps16*) possessed a single intron, while three genes (*ycf3*, *clpP*, and *rps12*) harbored two introns (Figure S2). Notably, *rps12* is the only trans-splicing gene, with its first exon situated in the LSC region, whereas the second and third exons reside in the IR regions. The phylogenetic results showed that *C. rosiflorus* was sister to *C. dammerii* with highly supported values (Figure 3 & Figure S3).

## Discussion and conclusions

In this study, we sequenced genome-skimming data and fully characterized the chloroplast genome of *C. rosiflorus*. The phylogenetic tree of *Cotoneaster* has been reconstructed, with the observed short branch lengths suggesting a recent and rapid evolutionary radiation within this genus, consistent with the findings reported by Meng et al. (2021). Intriguingly, the phylogenetic analysis uncovered a close relationship between *C. rosiflorus* and its sympatric species *C. dammerii*, which bears white flowers, rather than to species with pink flowers. Chloroplast capture events are notably frequent within Rosaceae and have been documented extensively (Liu et al. 2020; Meng et al. 2021; Chen et al. 2022). Thus, it is reasonable to speculate that *C. rosiflorus* captured the chloroplast from *C. dammerii*, potentially through hybridization and introgression events. Furthermore, eight species such as *C. vellaeus*, *C. cochleatus*, *C. microphyllus*, *C. affinis*, *C. gamblei*, *C. frigidus*, *C. dammerii*, and *C. vandelaarii* might also be hybrids (Meng et al. 2021). Next, we plan to enhance population samplings by including two additional sympatric species from this distribution area: *C. konishi*i, characterized by white petals, and *C. apiculatus*, known for its pink petals. Additionally, we will incorporate nuclear gene data to corroborate the aforementioned hypothesis. In conclusion, this study has greatly enriched our knowledge of the genetic makeup of *C. rosiflorus*, offering invaluable insights that contribute to a more comprehensive phylogenetic analysis of *Cotoneaster*.

## Supplementary Material

Supplementary materials.doc

Figure S2.tif

Figure S1.tif

Figure S3.tif

## Data Availability

The associated data of this study can be accessible in NCBI public database under the accession number OP800435. The corresponding BioProject, Bio-Sample, and SRA numbers are PRJNA899650, SAMN31665760, and SRR22254564.

## References

[CIT0001] Capella-Gutiérrez S, Silla-Martínez JM, Gabaldón T. 2009. trimAl: a tool for automated alignment trimming in large-scale phylogenetic analyses. Bioinformatics. 25(15):1972–1973. doi:10.1093/bioinformatics/btp348. 19505945 PMC2712344

[CIT0002] Chang KC, Wang CC, Deng SL, Kono Y, Lu FU, Peng CI. 2011. *Cotoneaster rosiflorus* (rosaceae), a new species from Taiwan. Bot Stud. 52(2):211–218.

[CIT0003] Chen SF, Milne R, Zhou RC, Meng KK, Yin QY, Guo W, Ma YP, Mao KS, Xu KW, Kim YD, et al. 2022. When tropical and subtropical congeners met: multiple ancient hybridization events within *Eriobotrya* in the Yunnan-Guizhou Plateau, a tropical-subtropical transition area in China. Mol Ecol. 31(5):1543–1561. doi:10.1111/mec.16325. 34910340

[CIT0004] Chen SF, Zhou YQ, Chen YR, Gu J. 2018. fastp: an ultra-fast all-in-one FASTQ preprocessor. Bioinformatics. 34(17):i884–i890. doi:10.1093/bioinformatics/bty560. 30423086 PMC6129281

[CIT0005] Dierckxsens N, Mardulyn P, Smits G. 2017. NOVOPlasty: de novo assembly of organelle genomes from whole genome data. Nucleic Acids Res. 45(4):e18. doi:10.1093/nar/gkw955. 28204566 PMC5389512

[CIT0006] Fryer J, Hylmö B. 2009. Cotoneasters: a comprehensive guide to shrubs for flowers, fruit, and foliage. Portland and London: Timber Press. p. 1–344.

[CIT0007] Hoang DT, Chernomor O, von Haeseler A, Minh BQ, Vinh LS. 2018. UFBoot2: improving the ultrafast bootstrap approximation. Mol Biol Evol. 35(2):518–522. doi:10.1093/molbev/msx281. 29077904 PMC5850222

[CIT0008] Li FF, Fan Q, Li QY, Chen SF, Guo W, Cui DF, Liao WB. 2014. Molecular phylogeny of *Cotoneaster* (Rosaceae) inferred from nuclear ITS and multiple chloroplast sequences. Plant Syst Evol. 300(6):1533–1546. doi:10.1007/s00606-014-0980-5.

[CIT0009] Liu BB, Hong DY, Zhou SL, Xu C, Dong WP, Johnson G, Wen J. 2019. *Phippsiomeles* and the resurrection of a redefined *Stranvaesia* in Maleae (Rosaceae). J. Syst. Evol. 57:6:678–694.

[CIT0010] Liu BB, Liu GN, Hong DY, Wen J. 2020. *Eriobotrya* belongs to *Rhaphiolepis* (Maleae, Rosaceae): evidence from chloroplast genome and nuclear ribosomal DNA data. Front Plant Sci. 10:1731. doi:10.3389/fpls.2019.01731. 32117331 PMC7019104

[CIT0011] Liu SY, Yang N, Li JL, Zhang XY, Yang HY, Chen HM, Liu C. 2023. CPGView: a package for visualizing detailed chloroplast genome structures. Mol. Ecol. Resour. 0:1–11. 10.1111/1755-0998.13729 36587992

[CIT0012] Meng KK, Chen SF, Xu KW, Zhou RC, Li MW, Dhamala MK, Liao WB, Fan Q. 2021. Phylogenomic analyses based on genome-skimming data reveal cyto-nuclear discordance in the evolutionary history of *Cotoneaster* (Rosaceae). Mol Phylogenet Evol. 158:107083. doi:10.1016/j.ympev.2021.107083. 33516804

[CIT0013] Minh BQ, Schmidt HA, Chernomor O, Schrempf D, Woodhams MD, von Haeseler A, Lanfear R. 2020. IQ-TREE 2: new models and efficient methods for phylogenetic inference in the genomic era. Mol Biol Evol. 37(5):1530–1534. doi:10.1093/molbev/msaa015. 32011700 PMC7182206

[CIT0014] Rozewicki J, Li S, Amada KM, Standley DM, Katoh K. 2019. MAFFT-DASH: integrated protein sequence and structural alignment. Nucleic Acids Res. 47(W1):W5–W10. doi:10.1093/nar/gkz342. 31062021 PMC6602451

[CIT0015] Shi LC, Chen HM, Jiang M, Wang LQ, Wu X, Huang LF, Liu C. 2019. CPGAVAS2, an integrated plastome sequence annotator and analyzer. Nucleic Acids Res. 47(W1):W65–W73. doi:10.1093/nar/gkz345. 31066451 PMC6602467

[CIT0016] Tillich M, Lehwark P, Pellizzer T, Ulbricht-Jones ES, Fischer A, Bock R, Greiner S. 2017. GeSeq-versatile and accurate annotation of organelle genomes. Nucleic Acids Res. 45(W1):W6–W11. doi:10.1093/nar/gkx391. 28486635 PMC5570176

