# Supplementary material for: The plastome and phylogenetic status of Cotoneaster rosiflorus (Rosaceae)
Source: Mitochondrial DNA B Resour. 2024 Aug 2;9(8):949–53. doi: 10.1080/23802359.2024.2385616 (PMC11299456; doi:10.1080/23802359.2024.2385616)
Supplement: Supplementary materials.doc [file TMDN_A_2385616_SM9732.doc]

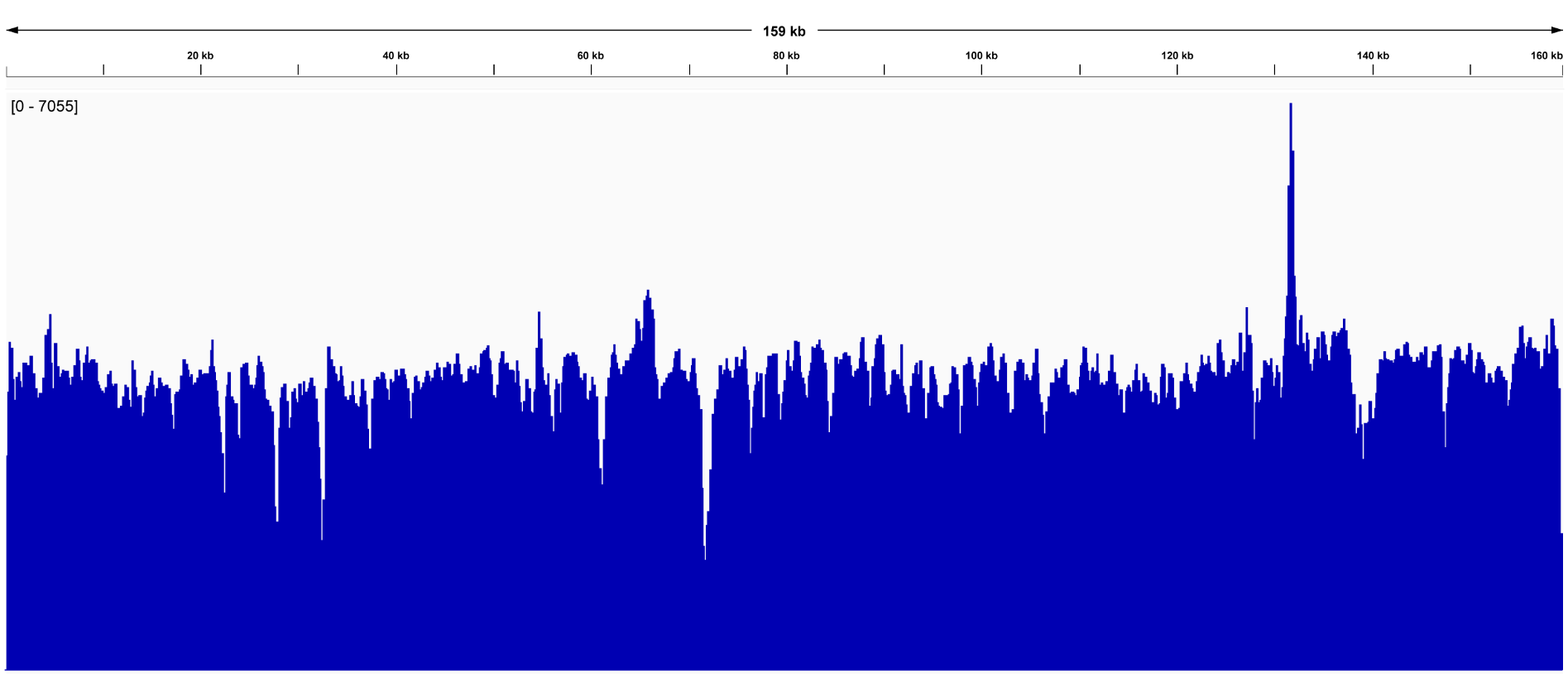


Figure S1. The overall coverage depth visualized in IGV (Robinson et al. 2011).


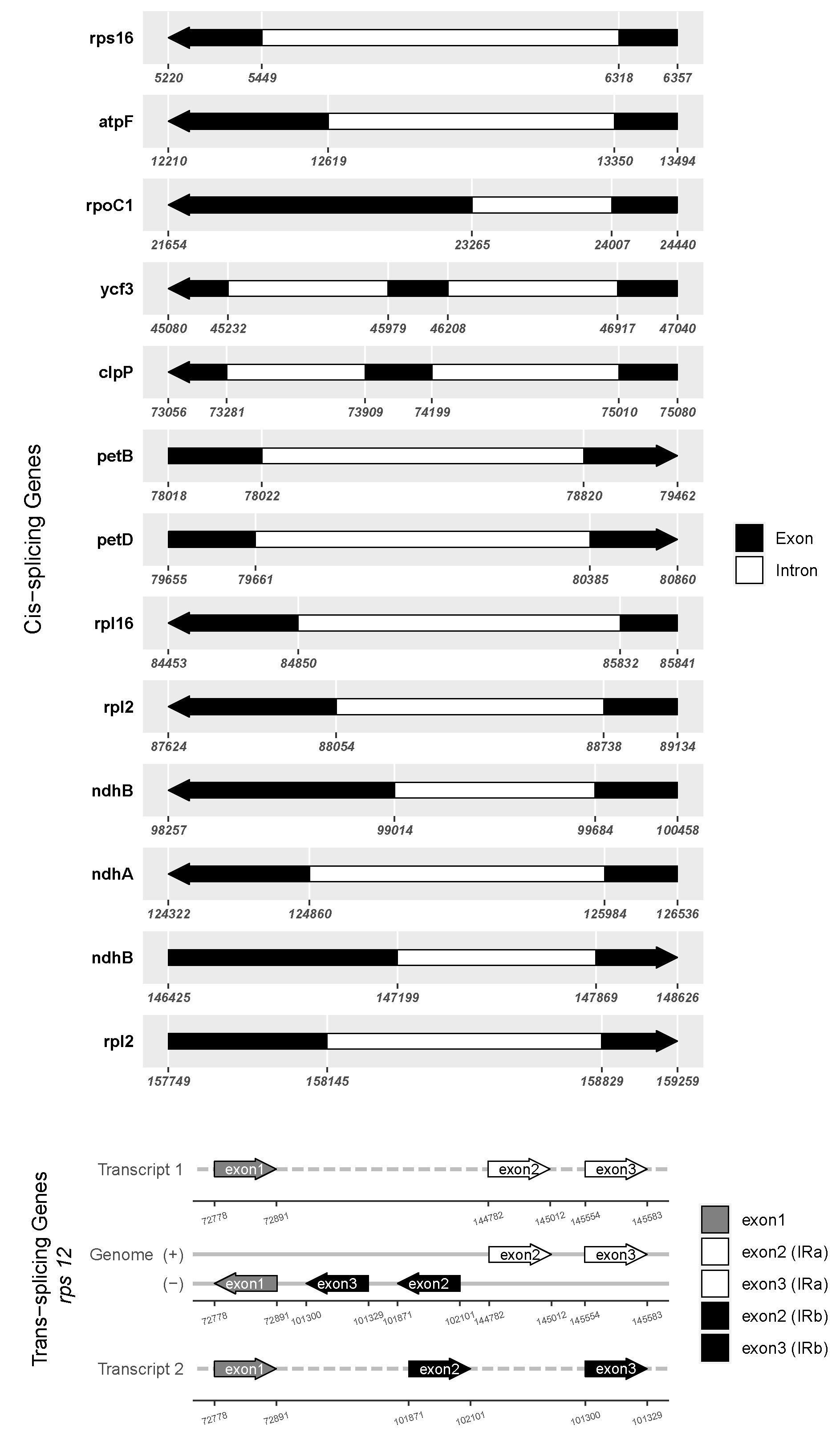


Figure S2. Cis-splicing and trans-splicing gene maps visualized in CPGView

(Liu et al. 2023).


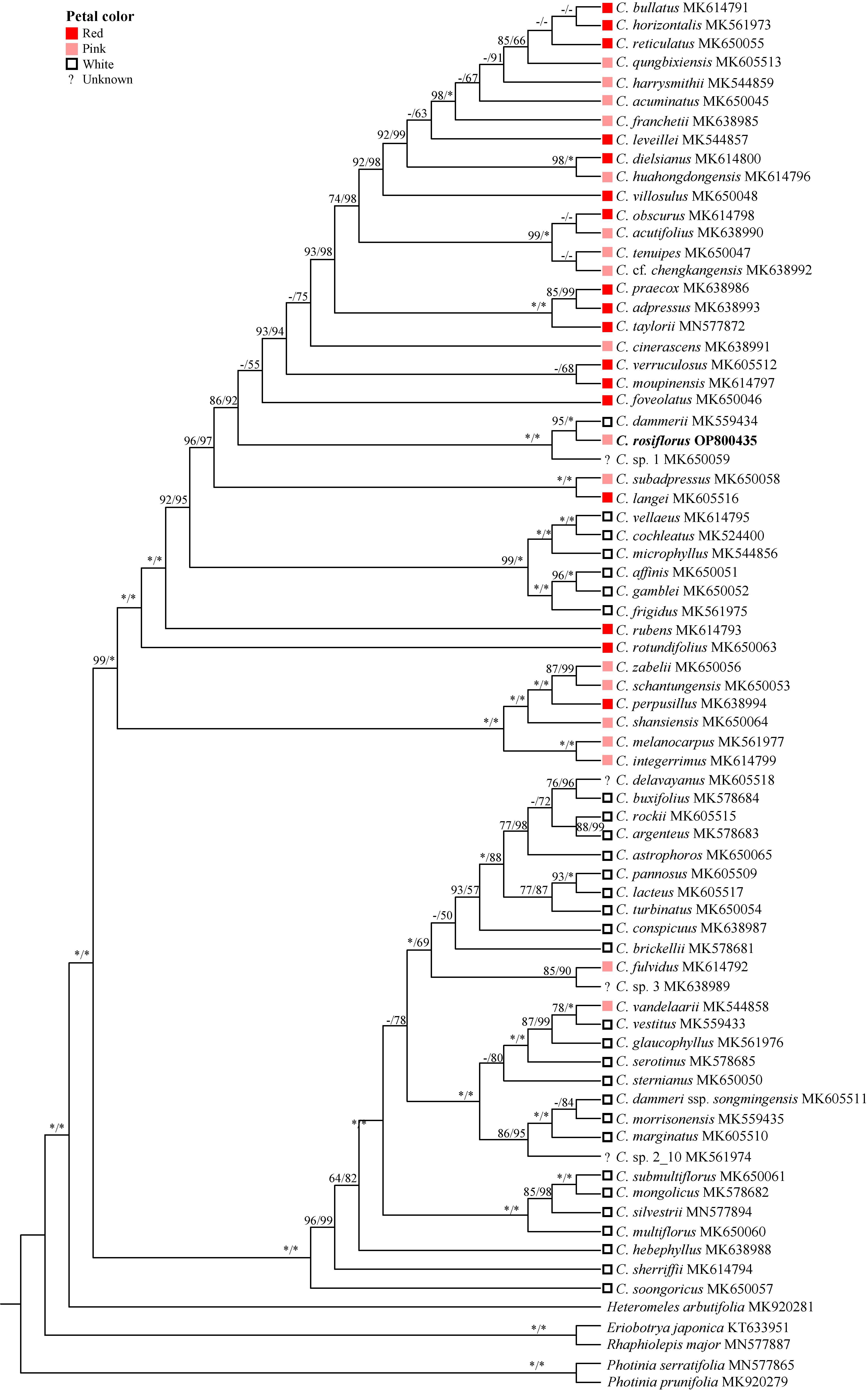


Figure S3. Maximum-likelihood phylogeny of *Cotoneaster*. Numbers indicate SH-aLRT/UFBS and values below 50 are indicated by “-”. Specifically, two sequences were downloaded from publicly available database based on Liu et al. (2019; MK920279 and MK920281), five sequences were obtained from Liu et al. (2020; MN577865, MN577872, MN577887, MN577894, and KT633951), and the remaining 69 sequences were sourced from Meng et al (2021).

References for supplementary materials:

Liu BB, Hong DY, Zhou SL, Xu C, Dong WP, Johnson G, Wen J. 2019. *Phippsiomeles* and the resurrection of a redefined *Stranvaesia* in Maleae (Rosaceae). J. Syst. Evol. 57, 6:678–694.

Liu BB, Liu GN, Hong DY, Wen J. 2020. *Eriobotrya* belongs to *Rhaphiolepis* (Maleae, Rosaceae): evidence from chloroplast genome and nuclear ribosomal DNA data. Front. Plant Sci. 10:1731.

Liu SY, Yang Ni, Li JL, Zhang XY, Yang HY, Chen HM, Liu C. 2023. CPGView: A package for visualizing detailed chloroplast genome structures. Mol. Ecol. Resour. 00, 1–11.

Meng KK, Chen SF, Xu KW, Zhou RC, Li MW, Dhamala MK, Liao WB, Fan Q. 2021. Phylogenomic analyses based on genome-skimming data reveal cyto-nuclear discordance in the evolutionary history of *Cotoneaster* (Rosaceae). Mol. Phylogenet. Evol. 158:107083.

Robinson JT, Thorvaldsdóttir H, Winckler W, Guttman M, Lander ES, Getz G, Mesirov JP. 2011. Integrative genomics viewer. Nat Biotechnol. 29(1):24–26.
