# Supplementary figures and images for: The plastome and phylogenetic status of Cotoneaster rosiflorus (Rosaceae)
Source: Mitochondrial DNA B Resour. 2024 Aug 2;9(8):949–53. doi: 10.1080/23802359.2024.2385616 (PMC11299456; doi:10.1080/23802359.2024.2385616)

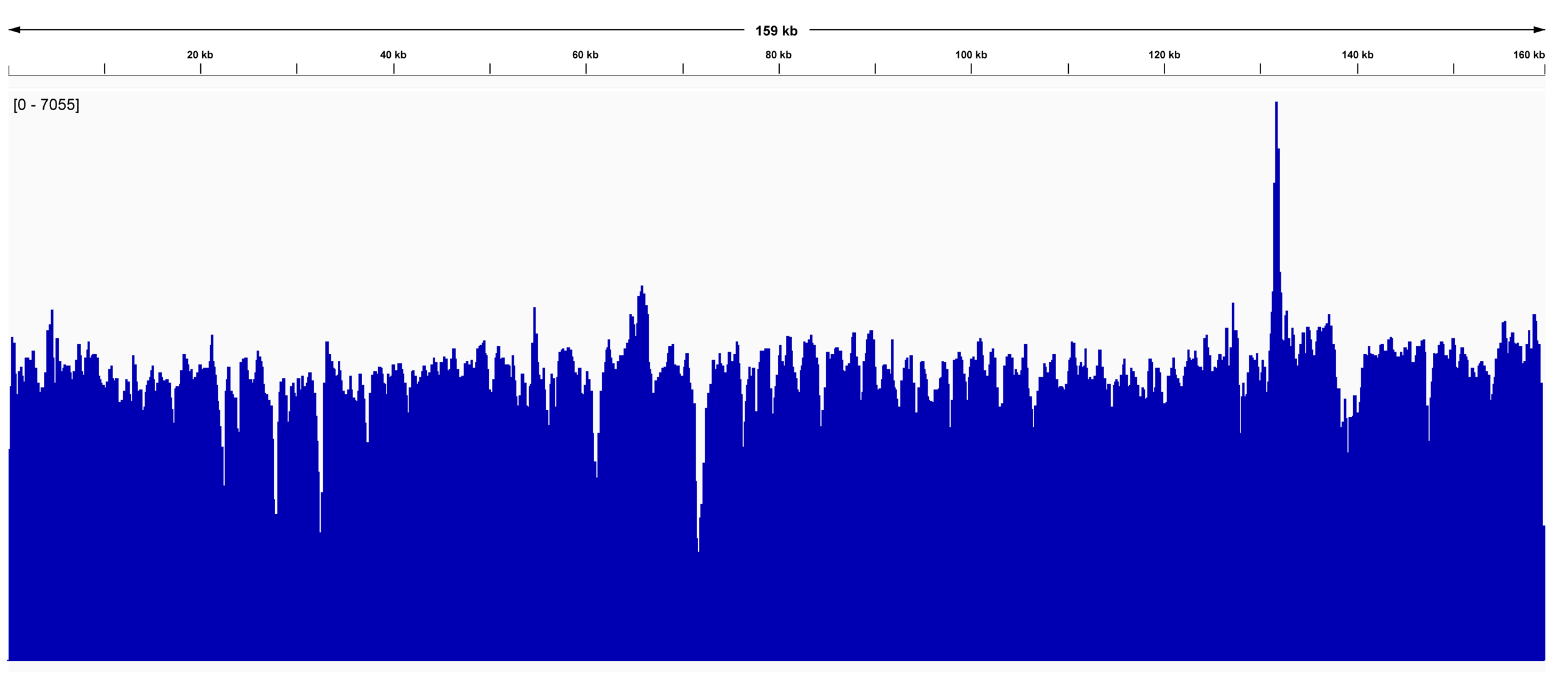

Supplement: Figure S1.tif [file TMDN_A_2385616_SM9730.tif]

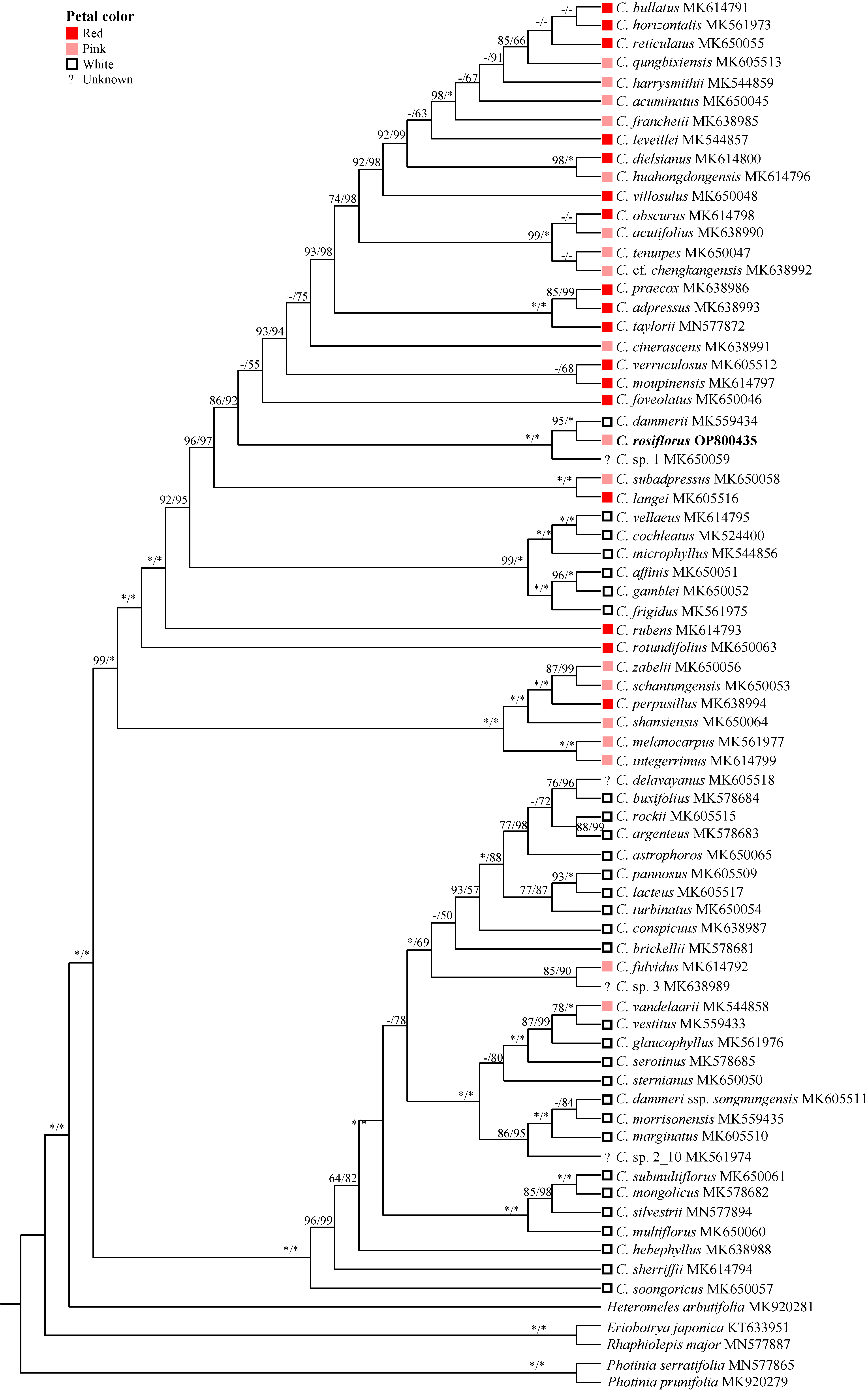

Supplement: Figure S3.tif [file TMDN_A_2385616_SM9729.tif]
